# Integrated Analysis of Transcriptomic and Genomic Data Reveals Blood Biomarkers With Diagnostic and Prognostic Potential in Non-small Cell Lung Cancer

**DOI:** 10.3389/fmolb.2022.774738

**Published:** 2022-03-04

**Authors:** Ibrahim H. Kaya, Olfat Al-Harazi, Mustafa T. Kaya, Dilek Colak

**Affiliations:** ^1^ College of Medicine, Alfaisal University, Riyadh, Saudi Arabia; ^2^ Department of Molecular Oncology, King Faisal Specialist Hospital and Research Centre, Riyadh, Saudi Arabia; ^3^ King Faisal School, Riyadh, Saudi Arabia

**Keywords:** lung cancer, NSCLC, early diagnosis, gene signature, blood, prognosis, biomarker, omics

## Abstract

**Background:** Lung cancer is the second most common cancer and the main leading cause of cancer-associated death worldwide. Non-small cell lung cancer (NSCLC) accounts for about 85% of lung cancer diagnoses and more than 50% of all lung cancer cases are diagnosed at an advanced stage; hence have poor prognosis. Therefore, it is important to diagnose NSCLC patients reliably and as early as possible in order to reduce the risk of mortality.

**Methods:** We identified blood-based gene markers for early NSCLC by performing a multi-omics approach utilizing integrated analysis of global gene expression and copy number alterations of NSCLC patients using array-based techniques. We also validated the diagnostic and the prognostic potential of the gene signature using independent datasets with detailed clinical information.

**Results:** We identified 12 genes that are significantly expressed in NSCLC patients’ blood, at the earliest stages of the disease, and associated with a poor disease outcome. We then validated 12-gene signature’s diagnostic and prognostic value using independent datasets of gene expression profiling of over 1000 NSCLC patients. Indeed, 12-gene signature predicted disease outcome independently of other clinical factors in multivariate regression analysis (HR = 2.64, 95% CI = 1.72–4.07; *p* = 1.3 × 10^−8^). Significantly altered functions, pathways, and gene networks revealed alterations in several key genes and cancer-related pathways that may have importance for NSCLC transformation, including *FAM83A*, *ZNF696*, *UBE2C*, *RECK*, *TIMM50, GEMIN7*, and *XPO5*.

**Conclusion:** Our findings suggest that integrated genomic and network analyses may provide a reliable approach to identify genes that are associated with NSCLC, and lead to improved diagnosis detecting the disease in early stages in patients’ blood instead of using invasive techniques and also have prognostic potential for discriminating high-risk patients from the low-risk ones.

## Introduction

Despite the advances in cancer therapies and raising awareness, lung cancer continues to be one of the most malignant tumors. It is the second most common cancer and the leading cause of cancer-related death worldwide (Bray et al., 2018). Non-small-cell lung carcinoma (NSCLC) is responsible for about 85% of lung cancers (Santarpia et al., 2015). The poor outcome of many NSCLC patients stems from the fact that many are diagnosed after their cancer has developed into advanced stages (Xie and Xie, 2019; Chen et al., 2020), further indicating the necessity of identifying NSCLC at an early stage for maximizing patient survival.

Recent genomic studies have shown that changes in gene expression and copy number variants (CNVs) have been associated with human diseases, including cancer (Colak et al., 2010; Colak et al., 2013), and identified potential biomarkers for the disease using RNA- or DNA-based approaches (Jabs et al., 2017; Chakraborty et al., 2018). Previous studies also indicated that integrated genomic and network-based analysis may lead to reliable biomarkers for human diseases (Jinhua Sheng et al., 2011; Colak et al., 2013; Al-Harazi et al., 2016; Chakraborty et al., 2018). However, most of the identified biomarkers requires invasive procedures or not able to diagnose the early NSCLC.

The aim of this study is to identify a blood-based gene signature potentially be involved in development of early stage of the disease and have a prognostic value. We performed integrated analysis of transcriptomic and genomic data to identify blood markers with diagnostic and prognostic potential in early NSCLC and validated its significance using over 1000 NSCLC patients from multiple independent genomic datasets with clinical data. The identified gene markers may improve the detection of diseases and help to develop therapeutic strategies.

## Materials and Methods

### Data Collection and the Integrated Analysis

**FIGURE 1:**
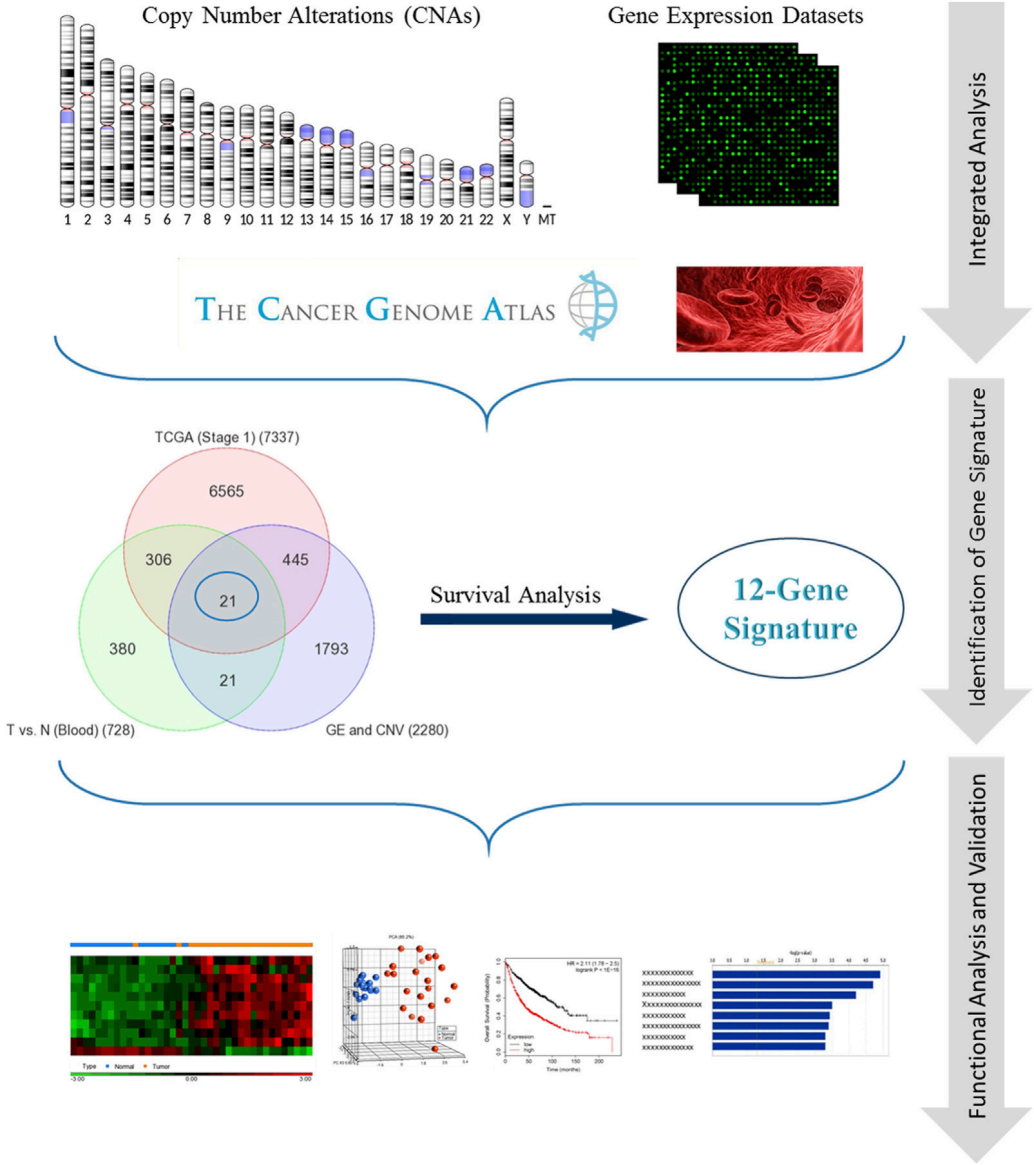
Schematic flowchart illustrating the methodology.

Whole-genome gene expression and copy number alterations (CNAs) datasets for 190 NSCLC patients were obtained from publicly available databases within NCBI GEO (www.ncbi.nlm.nih.gov/geo) (GSE37745 and GSE76730). These datasets were then analyzed as previously described (Jabs et al., 2017). Moreover, data for blood samples for lung cancer patients (*n* = 3) and controls (*n* = 3) were gathered from a publicly available database (GSE69732). Furthermore, we downloaded RNAseq dataset for NSCLC patients from The Cancer Genome Atlas (TCGA) that contains 576 samples (*n* = 517 tumor, 279 of which are with Stage 1 and 59 normal samples). We compared the transcriptome of early stage NSCLC (*n* = 279) with normal (*n* = 59) samples and identified the differentially expressed genes (DEGs). The DEGs were identified using Analysis of Variance (ANOVA) with adjusted *p*-value of <0.05 and absolute fold change (FC) ≥ 1.5. The *p* values were adjusted for multiple comparisons by false discovery rate (FDR) according to Benjamini–Hochberg step-up procedure (Benjamini and Hochberg, 1995). The integrated analysis was performed using the Venn diagram approach to identify the common DEGs among mRNA, CNA, early-stage NSCLC and blood gene expression datasets. We then identified genes that are significantly associated with patients’ survival by performing overall survival analysis for each gene separately on a dataset containing 1,144 lung cancer samples collected from 14 datasets (GSE4573 (Raponi et al., 2006), GSE14814 (Zhu et al., 2010), GSE8894 (Lee et al., 2008), GSE19188 (Hou et al., 2010), GSE3141 (Bild et al., 2006), GSE31210 (Yamauchi et al., 2012), GSE29013 (Xie et al., 2011), GSE37745 (Botling et al., 2013), caArray (Director’s Challenge Consortium for the Molecular Classification of Lung Adenocarcinoma et al., 2008), and TCGA (Cancer Genome Atlas Research Network, 2012)) (Győrffy et al., 2013). Figure 1 illustrates our methodology.

### Validation of the Diagnostic Value of the Gene Signature

For validating the diagnostic value of our gene signature, we used a TCGA dataset (*n* = 576) and an independent dataset from ArrayExpress (E-MTAB-5231). The independent dataset consists of 22 NSCLC samples and 17 normal adjacent controls. We performed unsupervised principal component analysis (PCA) and two-dimensional hierarchical clustering using PARTEK Genomics Suite (Partek Inc., St. Louis, MO, United States) for each dataset separately. Functional, pathway, and gene interaction network analyses of the gene signature were performed using QIAGEN’s Ingenuity Pathway Analysis (IPA®, QIAGEN Redwood City).

### Gene Ontology Enrichment, Pathway, and Gene Network Analyses

Gene ontology (GO) enrichment, pathway, and gene interaction network analyses were performed using (QIAGEN Inc., https://www.qiagenbioinformatics.com/products/ingenuity-pathway-analysis) and Database for Annotation, Visualization and Integrated Discovery (DAVID) (Dennis et al., 2003). We mapped the NSCLC-associated gene signature to its corresponding gene object in the Ingenuity pathway knowledge base and constructed the gene interaction networks. A right-tailed Fisher’s exact test was used to calculate a *p*-value determining the probability that the biological function (or pathway) assigned to the data set is explained by chance alone (Colak et al., 2020).

### Survival, Multivariate Analyses and NSCLC 12-Gene Classifier

Univariate and multivariate Cox regression analyses were used to assess our gene signature’s prognostic significance together with other clinical variables. We performed overall (OS) and progression free (PFS) survival on 1144 and 596 tumor samples, respectively. We calculated a 12-gene signature expression score for each patient that is average expression of up-regulated genes-average expression of down-regulated genes. We then used the median as the cutoff value for classifying patients into high and low risk groups. Survival curves were then plotted using the Kaplan-Meier method, and significance between survival curves was calculated by the log-rank test. In addition, multivariate analysis was performed using our 12-geneset taking histology (adenocarcinoma and squamous cell carcinoma), gender, and smoking history as covariates. A *p*-value < 0.05 was considered statistically significant.

Furthermore, we designed an NSCLC classifier using our 12-gene signature using several machine learning algorithms such as K-Nearest Neighbor, Linear Discriminant Analysis, Quadratic Discriminant Analysis, Nearest Centroid, and Support Vector Machine (SVM). We estimated the classification performance on TCGA with 10-fold cross validation. We utilized standardized gene expression levels of the 12-gene signature as feature values. Accuracy, specificity, sensitivity, and area under curve (AUC) were used statistics measures, as described previously (Al-Harazi et al., 2021a; Al-Harazi et al., 2021b). The Nearest Centroid algorithm with proportional prior probability has outperformed other algorithms. The analyses were performed using PARTEK Genomics Suite (Partek Inc., St. Lois, MO, United States).

## Results

### Identification of a Blood-Based Gene Signature for Early Stage Lung Cancer

We performed an integrated genomic analysis using four different transcriptomic and genomic datasets for human NSCLC. The analysis of transcriptomic and copy number alterations (CNAs) datasets (GSE37745 and GSE76730; 190 NSCLC) revealed 2,280 significantly expressed genes with copy number alterations (Jabs et al., 2017) (Figure 1). The analysis of whole-genome gene expression profiling of early stage NSCLC (*n* = 279) with normal (*n* = 59) samples revealed 7,337 genes (adjusted *p*-value <0.05 and fold change (FC) ≥ 1.5). Moreover, comparison of tumor transcriptome from patients’ blood with that of from normal controls resulted in 728 genes. We used Venn diagram approach to identify the common DEGs among mRNA, CNA, early-stage NSCLC and blood gene expression datasets that revealed 21 genes that are in common among all datasets (Figure 1). We then identified 12 genes (Table 1), defined as “12-gene signature,” that are significantly associated with patients’ survival by performing survival analysis of over 1,000 lung cancer samples (Figure 1).

**TABLE 1:** List of 12-gene signature that is identified in this study.

Gene	Gene Name	*p*-value	FC
*FAM83A*	family with sequence similarity 83, member A	1.82E-60	58.9
*GEMIN7*	gem (nuclear organelle) associated protein 7	4.56E-21	1.75
*ITPA*	inosine triphosphatase (nucleoside triphosphate pyrophosphatase)	3.50E-12	1.52
*NOP58*	NOP58 ribonucleoprotein	3.21E-28	1,67
*NR2C2AP*	nuclear receptor 2C2-associated protein	8.59E-25	1.84
*RECK*	reversion-inducing-cysteine-rich protein with kazal motifs	1.52E-41	-3.34
*TIMM50*	Translocase of inner mitochondrial membrane 50 homolog	3.19E-14	1.64
*TOMM40*	Translocase of outer mitochondrial membrane 40 homolog (yeast)	1.60E-13	1.60
*UBE2C*	ubiquitin-conjugating enzyme E2C	1.50E-42	12.5
*XPO5*	exportin 5	9.52E-31	1.97
*ZNF696*	zinc finger protein 696	3.76E-12	1.59
*ZNF7*	zinc finger protein 7	2.34E-19	1.62

**Abbreviations:** FC, fold change; FC, is calculated between the mean values of expression observed in tumor in comparison to normal using the data from The Cancer Genome Atlas (TCGA) (using Stage I only). Negative (−) value indicates downregulation.

### Diagnostic and Prognostic Significance of the 12-Gene Signature

**FIGURE 2:**
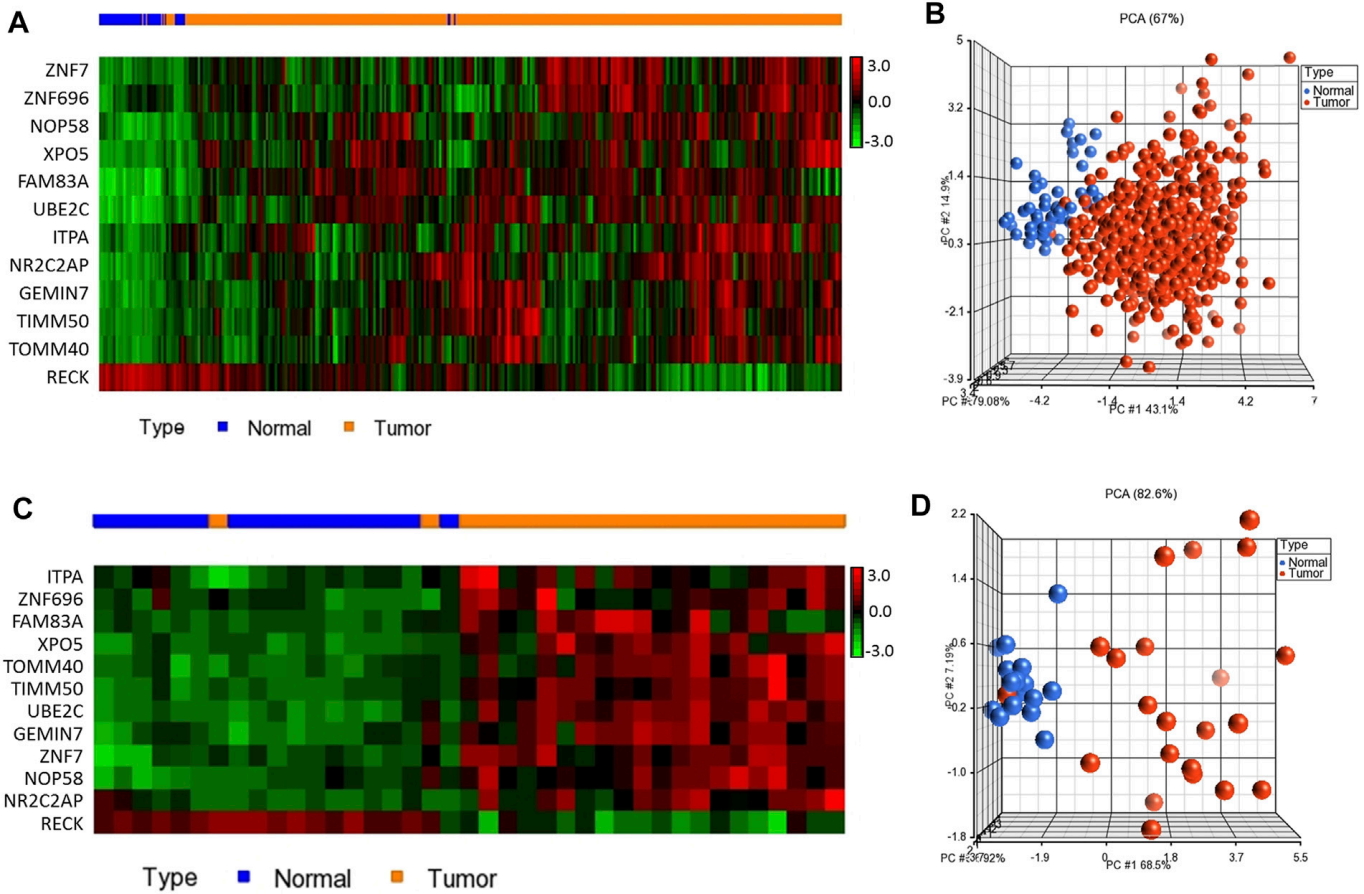
Two-dimensional hierarchical clustering using our gene signature clearly separated patients from normal controls in **(A)** TCGA (*n* = 576) and **(C)** E-MTAB-5231 (*n* = 39), respectively. The hierarchical clustering revealed two main clusters, one mainly composed of tumors and another composed of normal controls. Samples are denoted in columns and genes are denoted in rows. Unsupervised PCA for **(B)** TCGA (*n* = 576) and **(D)** E-MTAB-5231 (*n* = 39). Red indicates tumor and blue denotes normal samples.

To test the diagnostic value of the 12-gene list, we performed unsupervised two-dimensional hierarchical clustering and principal component analyses (PCA) on two datasets (TCGA, *n* = 576 and E-MTAB-5231, *n* = 39 samples). The unsupervised PCA and the two-dimensional hierarchical clustering clearly distinguished patients from normal control samples in both datasets (Figure 2).

**FIGURE 3:**
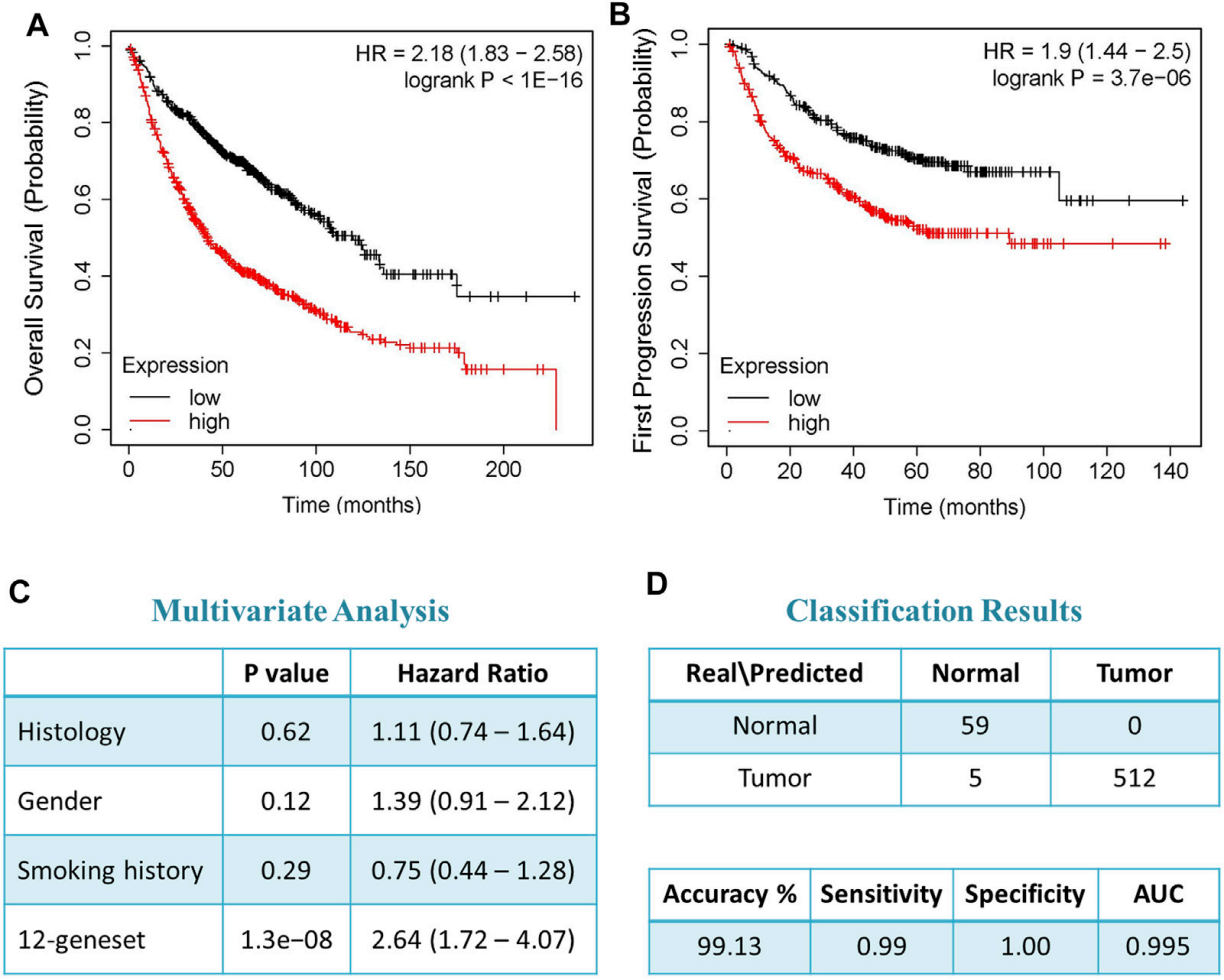
Prognostic significance of the 12-gene signature. **(A)** Overall and **(B)** progression free survival (PFS) analysis using NSCLC tumor samples (*n* = 1,144 samples). **(C)** Multivariate analysis using histology (adenocarcinoma and squamous cell carcinoma), gender, and smoking history as covariates. **(D)** Classification results of our gene signature using nearest centroid with proportional prior probability algorithm.

We confirmed the prognostic significance of our blood-based gene signature for overall as well as recurrence-free survival using a dataset with detailed clinical data from over 1000 NSCLC patients. The analysis demonstrated that high expression score based on 12-genes are significantly associated with poor disease outcome (Figures 3A,B). The 12-gene signature separated the patients into high risk and low-risk groups. Patients in the high-risk group had a significantly worse prognosis than the low-risk group with *p*-value < 1 × 10^−16^ (Figure 3). Patients in the high-risk group were more than twice likely to die from the disease than those in the low-risk group (Figure 3A). Similarly, the progression-free survival also showed that patients in the high-risk group had a poorer progression-free survival than patients in the low-risk group (Figure 3B).

Moreover, the multivariate analyses indicated that our 12-gene signature is prognosticating the outcome of the disease independent of other clinic-pathological variables, such as histology, smoking history, and gender (HR = 2.64, 95% CI = 1.72–4.07; *p*-value = 1.3 × 10^−8^) (Figure 3C). Furthermore, we designed the 12-gene classifier using nearest centroid with proportional prior probability algorithm that provided over 99% accuracy in classifying samples as tumors or normal controls (Figure 3D).

### Validation in Blood and Functional and Network Analyses

**FIGURE 4:**
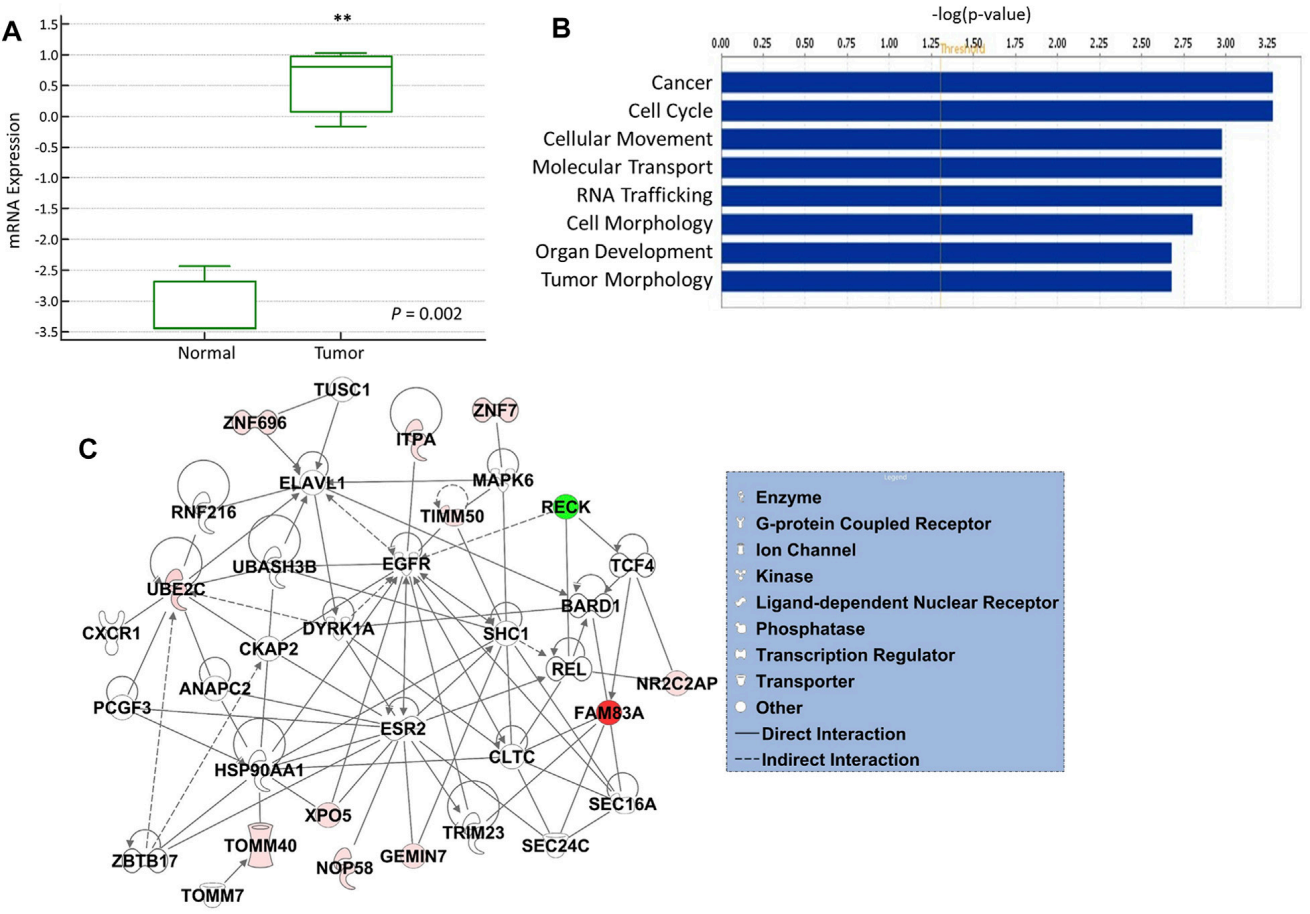
**(A)** mRNA gene expression of 12-gene signature score in blood from tumor vs. normal. **(B)** Gene ontology and functional analysis of the 12-gene signature. X-axis represents the significance (–log10 (*p*-value)) of the functional term. *p*-value of 0.05 is indicated as the threshold line in the figure **(C)** Gene interaction network analyses of the 12-gene signature. Red/green indicates higher/lower expression in NSCLC compared to controls. Straight lines are for direct interactions and dashed lines for indirect ones.

The expression of 12-gene in blood samples from patients and healthy controls (GSE69732) were compared that revealed that 12-gene signature score is significantly higher in tumor compared to normal (*p*-value = 0.002, Figure 4A). Functional and gene network analyses of the gene signature were performed using IPA which indicated that 12 genes were significantly associated with cancer, cell cycle, cellular movement, molecular transport, RNA trafficking, cell morphology, organ development, and tumor morphology (Figure 4B). Moreover, gene interaction networks revealed several key genes and cancer-related pathways that may role for early NSCLC transformation and disease progression, including *FAM83A*, *ZNF696*, *UBE2C*, *RECK*, *TIMM50*, *GEMIN7*, and *XPO5* (Figure 4C).

## Discussion

In this study, we sought to identify blood-based biomarkers with diagnostic and prognostic potential for early lung cancer using integrated analysis of multiple high dimensional independent datasets of transcriptomic and genomic datasets that detect the disease in early stages in patients’ biological fluids rather than using invasive techniques.

We identified 12-gene signature using integrated omics approach and validated its diagnostic and prognostic significance for overall and recurrence-free survival using data from over 1000 lung cancer patients’ samples with detailed clinical data. The analysis demonstrated that high 12-gene signature score was significantly associated with poor disease outcome. Previous studies reported that the integrated analysis of transcriptomic and genomic data may lead to reliable biomarkers that are more robust in disease classification and may have role in tumorigenesis (Colak et al., 2010; Al-Harazi et al., 2016; Chakraborty et al., 2018; Al-Harazi et al., 2021b). Indeed, several potential cancer driver genes that are involved in tumor initiation and progression have been identified using this approach (Colak et al., 2010; Colak et al., 2013; Ohshima et al., 2017).

Functional, pathway, and gene network analyses revealed significant biological functions, including cancer, cell cycle, cellular movement, molecular transport, and RNA trafficking, as well as several key genes and cancer-related pathways that may have importance for NSCLC transformation, including *FAM83A*, *ZNF696*, *UBE2C*, *RECK*, *TIMM50, GEMIN7*, and *XPO5*. Indeed, some of the identified genes were reported to be associated with cancers, including lung cancer. For example, *FAM83A* was found to be highly expressed in lung tumors (Li et al., 2015; Snijders et al., 2017). *RECK* is downregulated in esophageal squamous cell carcinoma (ESCC) and associated with a poor survival in ESCC (Zhu et al., 2017). The *UBE2C* gene is overexpressed in different types of cancers and considered a new target for cancers therapies (Dastsooz et al., 2019). Moreover, we used a machine learning algorithm to develop a model using our 12-gene signature for performing classification and tested its classification accuracy using over 500 lung cancer patients’ data that resulted in 99% prediction accuracy.

In conclusion, the 12-gene signature that we identified in this study reveals several genes and pathways that may be essential for early NSCLC transformation and progression and has potential to detect the disease in patients’ blood instead of utilizing invasive techniques. The integrated omics and network analyses may lead to robust biomarkers for the detection of early lung cancer and may lead to improved diagnosis, prognosis and therapeutic options.

## Data Availability

Publicly available datasets were analyzed in this study. These data can be found here: The Cancer Genome Atlas (TCGA), ArrayExpress, and the NCBI Gene Expression Omnibus.

## References

[B1] Al-Harazi O. Al Insaif S. Al-Ajlan M. A. Kaya N. Dzimiri N. Colak D. (2016). Integrated Genomic and Network-Based Analyses of Complex Diseases and Human Disease Network. J. Genet. Genomics 43 (6), 349–367. 10.1016/j.jgg.2015.11.002 27318646

[B2] Al-Harazi O. Kaya I. H. Al-Eid M. Alfantoukh L. Al Zahrani A. S. Al Sebayel M. (2021). Identification of Gene Signature as Diagnostic and Prognostic Blood Biomarker for Early Hepatocellular Carcinoma Using Integrated Cross-Species Transcriptomic and Network Analyses. Front. Genet. 12, 710049. 10.3389/fgene.2021.710049 34659334 PMC8511318

[B3] Al-Harazi O. Kaya I. H. El Allali A. Colak D. (2021). A Network-Based Methodology to Identify Subnetwork Markers for Diagnosis and Prognosis of Colorectal Cancer. Front. Genet. 12, 721949. 10.3389/fgene.2021.721949 34790220 PMC8591094

[B4] Benjamini Y. Hochberg Y. (1995). Controlling the False Discovery Rate: a Practical and Powerful Approach to Multiple Testing. J. R. Stat. Soc. Ser. B (Methodological) 57, 289–300. 10.1111/j.2517-6161.1995.tb02031.x

[B5] Bild A. H. Yao G. Chang J. T. Wang Q. Potti A. Chasse D. (2006). Oncogenic Pathway Signatures in Human Cancers as a Guide to Targeted Therapies. Nature 439 (7074), 353–357. 10.1038/nature04296 16273092

[B6] Botling J. Edlund K. Lohr M. Hellwig B. Holmberg L. Lambe M. (2013). Biomarker Discovery in Non-small Cell Lung Cancer: Integrating Gene Expression Profiling, Meta-Analysis, and Tissue Microarray Validation. Clin. Cancer Res. 19 (1), 194–204. 10.1158/1078-0432.CCR-12-1139 23032747

[B7] Bray F. Ferlay J. Soerjomataram I. Siegel R. L. Torre L. A. Jemal A. (2018). Global Cancer Statistics 2018: GLOBOCAN Estimates of Incidence and Mortality Worldwide for 36 Cancers in 185 Countries. CA: A Cancer J. Clinicians 68 (6), 394–424. 10.3322/caac.21492 30207593

[B8] Cancer Genome Atlas Research Network (2012). Comprehensive Genomic Characterization of Squamous Cell Lung Cancers. Nature 489 (7417), 519–525. 10.1038/nature11404 22960745 PMC3466113

[B9] Chakraborty S. Hosen M. I. Ahmed M. Shekhar H. U. (2018). Onco-Multi-OMICS Approach: A New Frontier in Cancer Research. Biomed. Res. Int. 2018, 1–14. 10.1155/2018/9836256 PMC6192166 30402498

[B10] Chen R. Manochakian R. James L. Azzouqa A.-G. Shi H. Zhang Y. (2020). Emerging Therapeutic Agents for Advanced Non-small Cell Lung Cancer. J. Hematol. Oncol. 13 (1), 58. 10.1186/s13045-020-00881-7 32448366 PMC7245927

[B11] Colak D. Al-Harazi O. Mustafa O. M. Meng F. Assiri A. M. Dhar D. K. (2020). RNA-seq Transcriptome Profiling in Three Liver Regeneration Models in Rats: Comparative Analysis of Partial Hepatectomy, ALLPS, and PVL. Sci. Rep. 10 (1), 5213. 10.1038/s41598-020-61826-1 32251301 PMC7089998

[B12] Colak D. Chishti M. A. Al-Bakheet A.-B. Al-Qahtani A. Shoukri M. M. Goyns M. H. (2010). Integrative and Comparative Genomics Analysis of Early Hepatocellular Carcinoma Differentiated from Liver Regeneration in Young and Old. Mol. Cancer 9, 146. 10.1186/1476-4598-9-146 20540791 PMC2898705

[B13] Colak D. Nofal A. Albakheet A. Nirmal M. Jeprel H. Eldali A. (2013). Age-specific Gene Expression Signatures for Breast Tumors and Cross-Species Conserved Potential Cancer Progression Markers in Young Women. PLoS One 8 (5), e63204. 10.1371/journal.pone.0063204 23704896 PMC3660335

[B14] Dastsooz H. Cereda M. Donna D. Oliviero S. (2019). A Comprehensive Bioinformatics Analysis of UBE2C in Cancers. Ijms 20 (9), 2228. 10.3390/ijms20092228 PMC6539744 31067633

[B15] DennisG.Jr.ShermanB. T.HosackD. A.YangJ.GaoW.LaneH. C. (2003). DAVID: Database for Annotation, Visualization, and Integrated Discovery. Genome Biol. 4 (5), P3. 10.1186/gb-2003-4-5-p3 12734009

[B16] Director's Challenge Consortium for the Molecular Classification of Lung Adenocarcinoma Shedden K. Taylor J. M. Enkemann S. A. Tsao M. S. Yeatman T. J. (2008). Gene Expression-Based Survival Prediction in Lung Adenocarcinoma: a Multi-Site, Blinded Validation Study. Nat. Med. 14 (8), 822–827. 10.1038/nm.1790 18641660 PMC2667337

[B17] Győrffy B. Surowiak P. Budczies J. Lánczky A. (2013). Online Survival Analysis Software to Assess the Prognostic Value of Biomarkers Using Transcriptomic Data in Non-small-cell Lung Cancer. PLoS One 8 (12), e82241. 10.1371/journal.pone.0082241 24367507 PMC3867325

[B18] Hou J. Aerts J. den Hamer B. van Ijcken W. den Bakker M. Riegman P. (2010). Gene Expression-Based Classification of Non-small Cell Lung Carcinomas and Survival Prediction. PLoS One 5 (4), e10312. 10.1371/journal.pone.0010312 20421987 PMC2858668

[B19] Jabs V. Edlund K. König H. Grinberg M. Madjar K. Rahnenführer J. (2017). Integrative Analysis of Genome-wide Gene Copy Number Changes and Gene Expression in Non-small Cell Lung Cancer. PLoS One 12 (11), e0187246. 10.1371/journal.pone.0187246 29112949 PMC5675410

[B20] Jinhua Sheng J. Hong-Wen Deng H. W. Calhoun V. D. Yu-Ping Wang Y. P. (2011). Integrated Analysis of Gene Expression and Copy Number Data on Gene Shaving Using Independent Component Analysis. Ieee/acm Trans. Comput. Biol. Bioinf. 8 (6), 1568–1579. 10.1109/TCBB.2011.71 PMC3146966 21519112

[B21] Lee E.-S. Son D.-S. Kim S.-H. Lee J. Jo J. Han J. (2008). Prediction of Recurrence-free Survival in Postoperative Non-small Cell Lung Cancer Patients by Using an Integrated Model of Clinical Information and Gene Expression. Clin. Cancer Res. 14 (22), 7397–7404. 10.1158/1078-0432.CCR-07-4937 19010856

[B22] Li Y. Xiao X. Ji X. Liu B. Amos C. I. (2015). RNA-seq Analysis of Lung Adenocarcinomas Reveals Different Gene Expression Profiles between Smoking and Nonsmoking Patients. Tumor Biol. 36 (11), 8993–9003. 10.1007/s13277-015-3576-y PMC4674426 26081616

[B23] Ohshima K. Hatakeyama K. Nagashima T. Watanabe Y. Kanto K. Doi Y. (2017). Integrated Analysis of Gene Expression and Copy Number Identified Potential Cancer Driver Genes with Amplification-dependent Overexpression in 1,454 Solid Tumors. Sci. Rep. 7 (1), 641. 10.1038/s41598-017-00219-3 28377632 PMC5428069

[B24] Raponi M. Zhang Y. Yu J. Chen G. Lee G. Taylor J. M. G. (2006). Gene Expression Signatures for Predicting Prognosis of Squamous Cell and Adenocarcinomas of the Lung. Cancer Res. 66 (15), 7466–7472. 10.1158/0008-5472.CAN-06-1191 16885343

[B25] Santarpia M. González-Cao M. Viteri S. Karachaliou N. Altavilla G. Rosell R. (2015). Programmed Cell Death Protein-1/programmed Cell Death Ligand-1 Pathway Inhibition and Predictive Biomarkers: Understanding Transforming Growth Factor-Beta Role. Transl Lung Cancer Res. 4 (6), 728–742. 10.3978/j.issn.2218-6751.2015.12.04 26798582 PMC4700220

[B26] Snijders A. M. Lee S.-Y. Hang B. Hao W. Bissell M. J. Mao J.-H. (2017). FAM83 Family Oncogenes Are Broadly Involved in Human Cancers: an Integrative Multi-Omics Approach. Mol. Oncol. 11 (2), 167–179. 10.1002/1878-0261.12016 28078827 PMC5527452

[B27] Xie H. Xie C. (2019). A Six-Gene Signature Predicts Survival of Adenocarcinoma Type of Non-small-cell Lung Cancer Patients: A Comprehensive Study Based on Integrated Analysis and Weighted Gene Coexpression Network. Biomed. Res. Int. 2019, 1–16. 10.1155/2019/4250613 PMC6925693 31886214

[B28] Xie Y. Xiao G. Coombes K. R. Behrens C. Solis L. M. Raso G. (2011). Robust Gene Expression Signature from Formalin-Fixed Paraffin-Embedded Samples Predicts Prognosis of Non-small-cell Lung Cancer Patients. Clin. Cancer Res. 17 (17), 5705–5714. 10.1158/1078-0432.CCR-11-0196 21742808 PMC3166982

[B29] Yamauchi M. Yamaguchi R. Nakata A. Kohno T. Nagasaki M. Shimamura T. (2012). Epidermal Growth Factor Receptor Tyrosine Kinase Defines Critical Prognostic Genes of Stage I Lung Adenocarcinoma. PLoS One 7 (9), e43923. 10.1371/journal.pone.0043923 23028479 PMC3446964

[B30] Zhu C.-Q. Ding K. Strumpf D. Weir B. A. Meyerson M. Pennell N. (2010). Prognostic and Predictive Gene Signature for Adjuvant Chemotherapy in Resected Non-small-cell Lung Cancer. Jco 28 (29), 4417–4424. 10.1200/JCO.2009.26.4325 PMC2988634 20823422

[B31] Zhu J. Ling Y. Xu Y. Lu M. Liu Y. Zhang C. (2017). Promoter Hypermethylation of the RECK Gene Is Associated with its Low Expression and Poor Survival of Esophageal Squamous Cell Carcinoma. Oncol. Lett. 13 (3), 1911–1918. 10.3892/ol.2017.5656 28454343 PMC5403254

